# Supplementary figures and images for: Single cell transcriptomics identifies distinct choroid cell populations involved in visually guided eye growth
Source: Front Ophthalmol (Lausanne). 2023 Oct 6;3:1245891. doi: 10.3389/fopht.2023.1245891 (PMC10883300; doi:10.3389/fopht.2023.1245891)

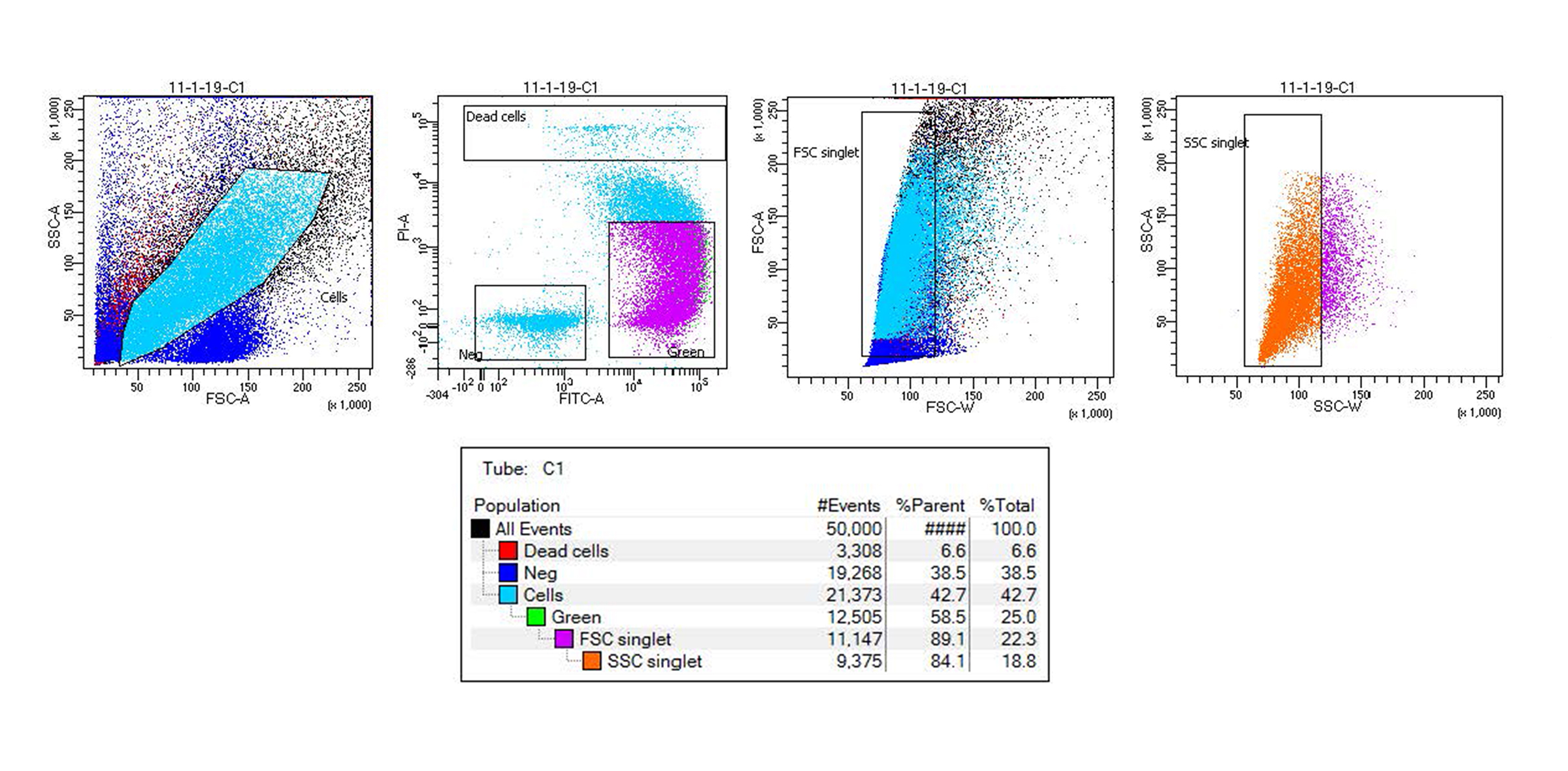

Supplement: Supplementary Figure 1 — FACS gating strategy for isolation of choroidal cells. FACS plot of all events (black) with forward scatter area intensity (FSC-A) and side scatter area intensity (SSC-A) containing a gate to select only single cells (royal blue) followed by a FACS plot based on green fluorescence intensity (FITC-A) and red fluorescence intensity (PI-A). Living cells labelled with calcein AM (green), but not with EthDIII (red; dead cells) were backgated onto gates of forward scatter width intensity (FSC-W) and forward scatter area intensity (FSC-A) followed by gates of side scatter width intensity (SSC-W) and side scatter area intensity (SSC-A) to select only single living cells while avoiding cell aggregates. [file Image_1.tif]

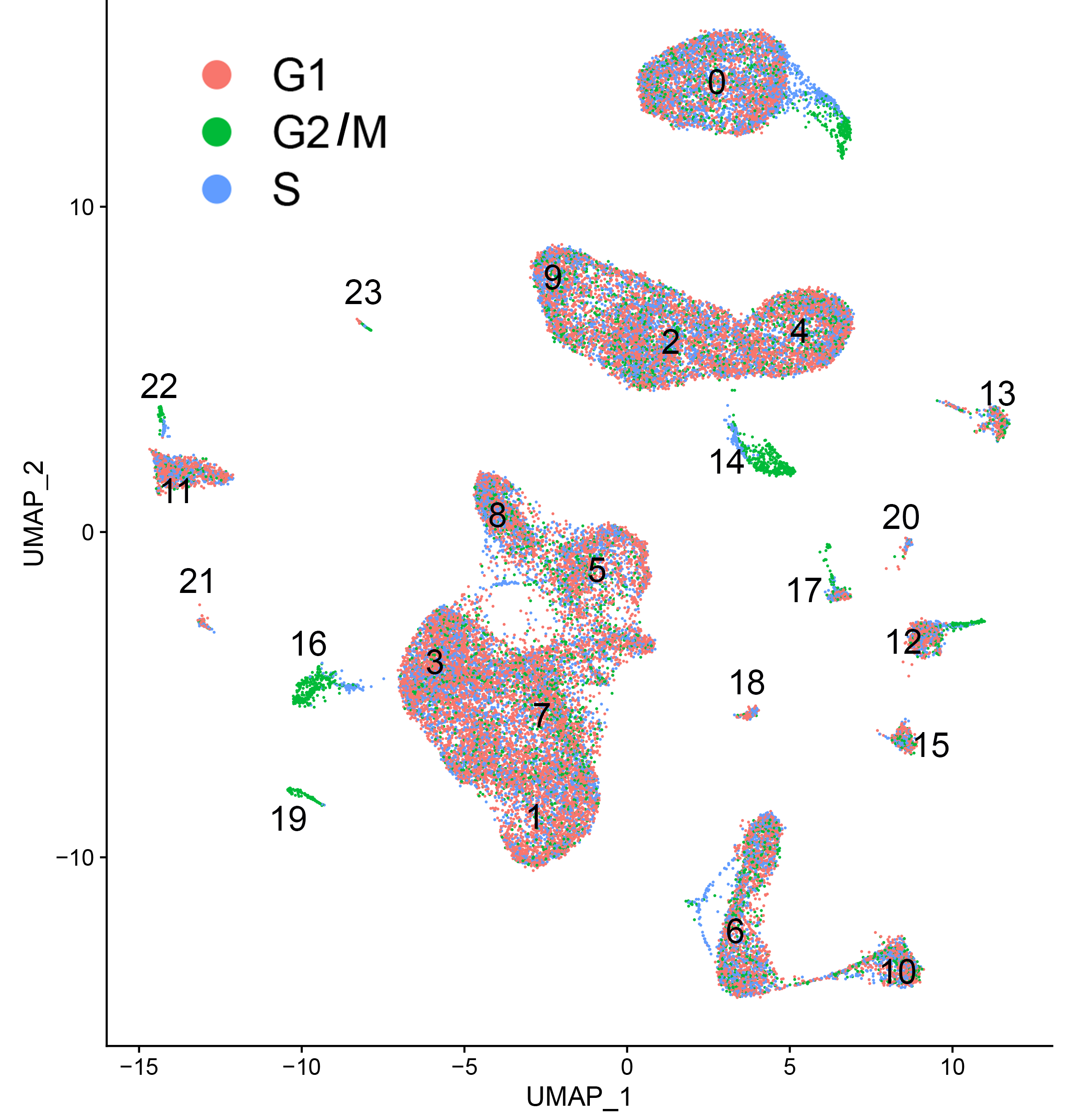

Supplement: Supplementary Figure 2 — Dot blot showing the expression pattern and level of some established marker genes (shown in columns) for each major cell type (shown in rows) in chicken choroid. Color intensities show the expression level of the indicated gene. [file Image_2.tif]

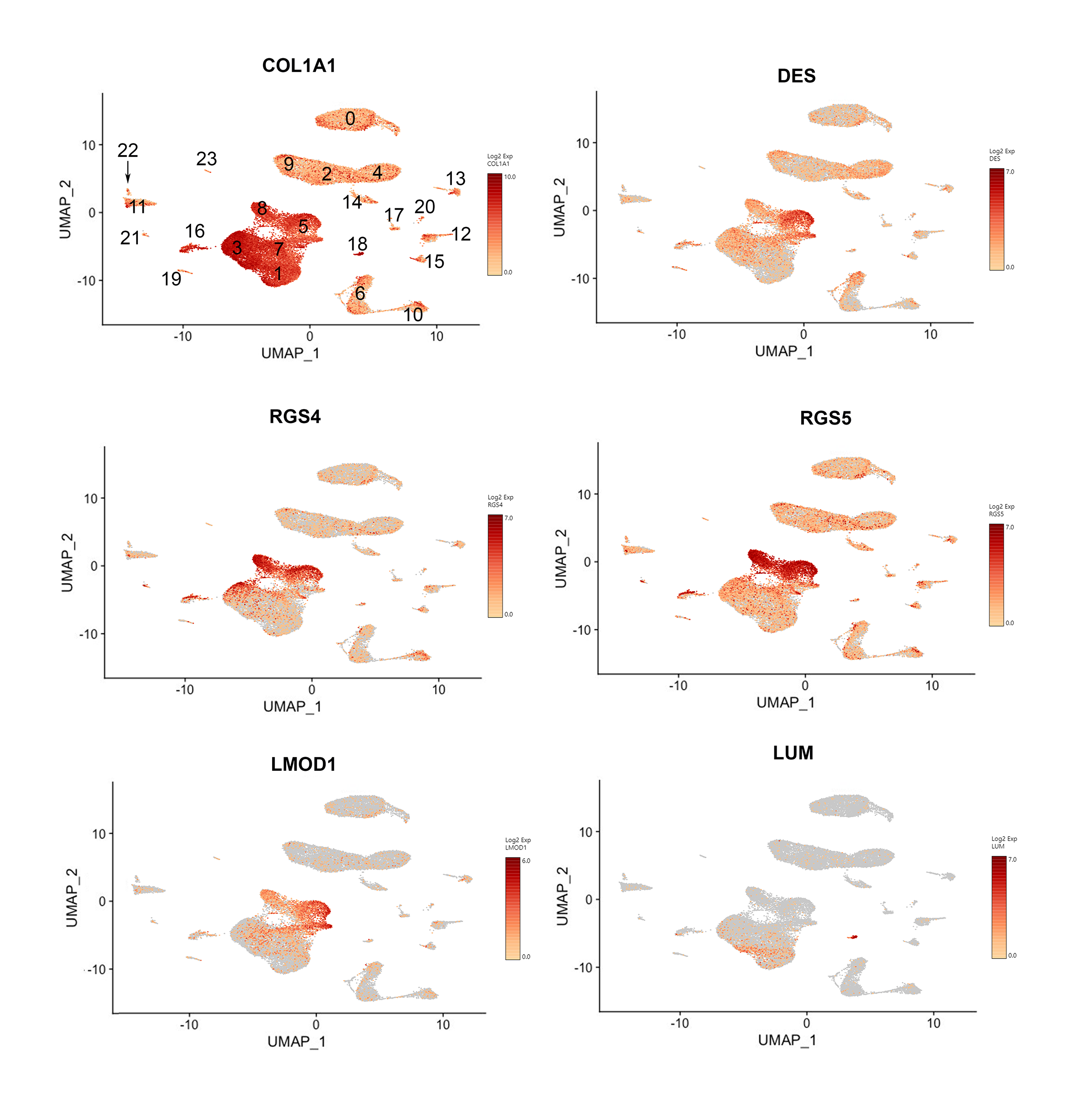

Supplement: Supplementary Figure 3 — Uniform Manifold Approximation and Projection (UMAP) maps showing phases of cell cycle assigned for each of the 24 choroid cell clusters as determined from the “Cell Cycle Scoring” function in Seurat v 4.0.6). G1, growth 1 phase; G2/M, growth 2/mitosis phase; S, synthesis phase. [file Image_3.tif]

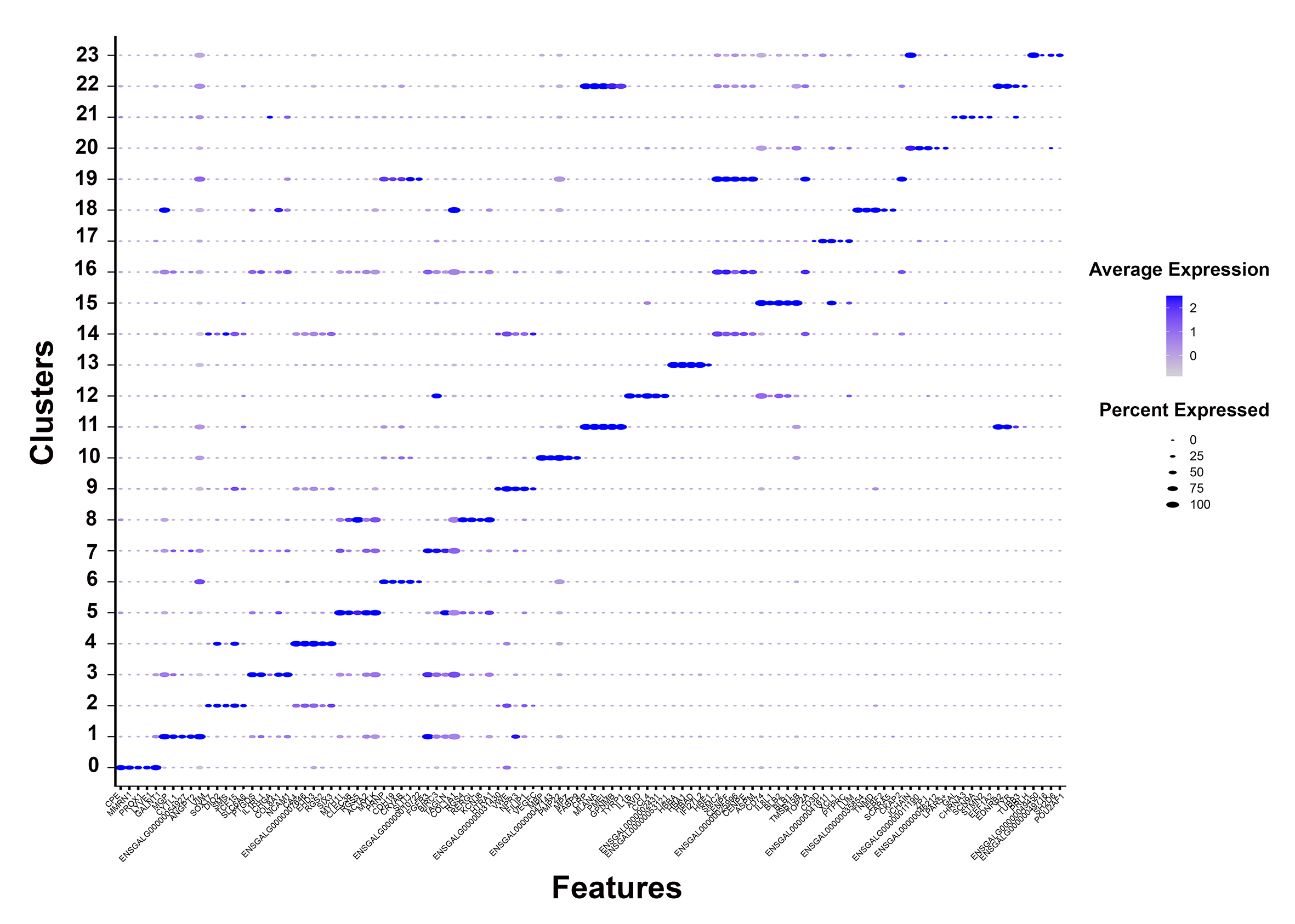

Supplement: Supplementary Figure 4 — Uniform Manifold Approximation and Projection (UMAP) maps showing expression of fibroblast marker genes in choroidal cell populations. Col1A1, collagen type I alpha 1 chain; DES, desmin; RGS4, regulator of G protein signaling 4; RGS5, regulator of G protein signaling 5; LMOD1, leiomodin 1; LUM, lumican. [file Image_4.tif]

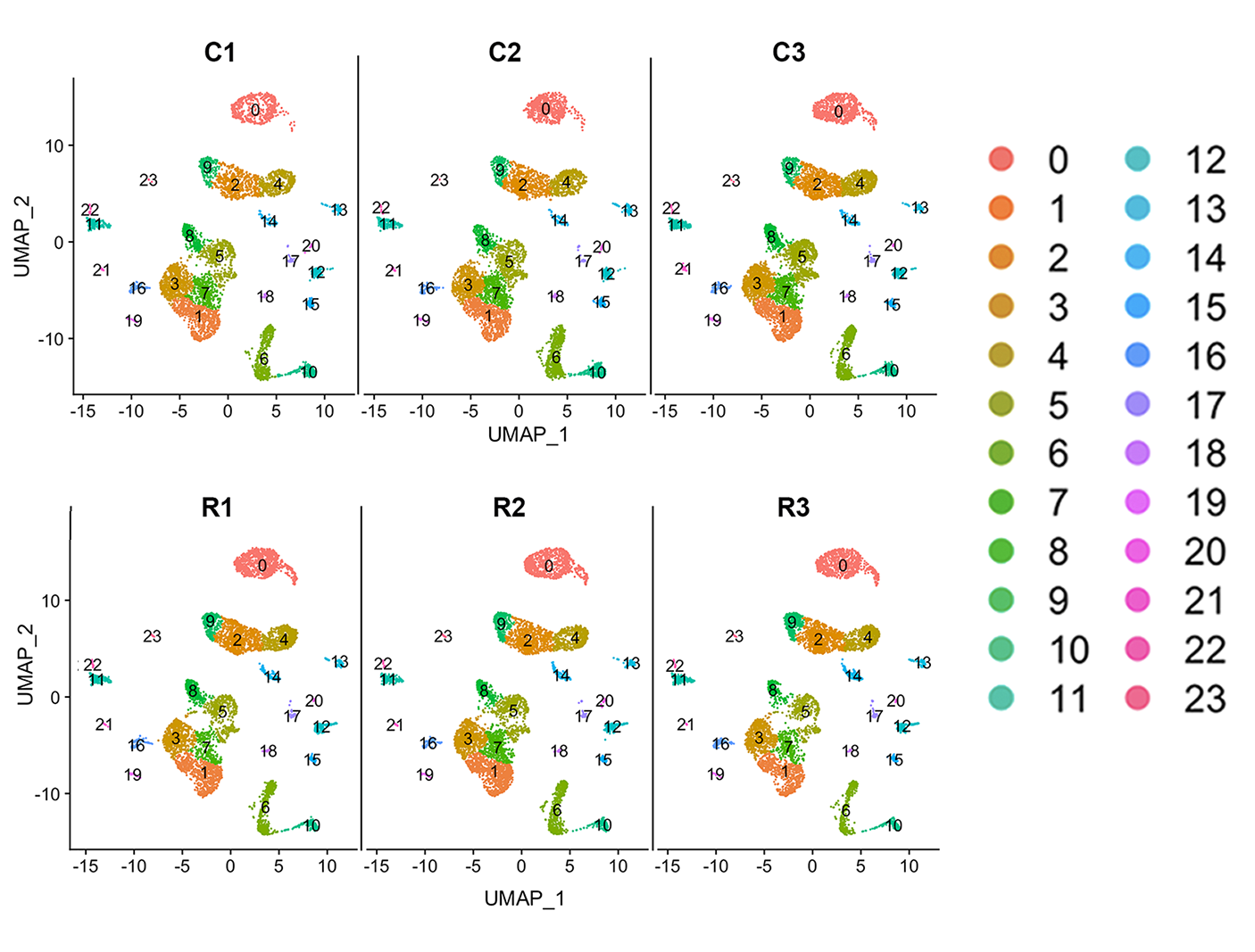

Supplement: Supplementary file 5 [file Image_5.tif]
