# Supplementary material for: Single cell transcriptomics identifies distinct choroid cell populations involved in visually guided eye growth
Source: Front Ophthalmol (Lausanne). 2023 Oct 6;3:1245891. doi: 10.3389/fopht.2023.1245891 (PMC10883300; doi:10.3389/fopht.2023.1245891)
Supplement: Supplementary file 6 [file Table_1.docx]

| **Sample** | **# Cells/Sample** | **Mean Reads per Cell** | **Median Genes per Cell** |
| --- | --- | --- | --- |
| C1 | 10,221 | 46,031 | 1,110 |
| C2 | 11,251 | 43,351 | 1,095 |
| C3 | 12,469 | 41,847 | 1,225 |
| R1 | 11,976 | 35,621 | 1,080 |
| R2 | 12,392 | 40,631 | 1,140 |
| R3 | 12,745 | 43,480 | 1,123 |

**Table S1.** Sample data for Single Cell RNA sequencing.
